# Correlations between immune response and vascularization qRT-PCR gene expression clusters in squamous cervical cancer

**DOI:** 10.1186/s12943-015-0350-0

**Published:** 2015-03-31

**Authors:** Simone Punt, Jeanine J Houwing-Duistermaat, Iris A Schulkens, Victor L Thijssen, Elisabeth M Osse, Cornelis D de Kroon, Arjan W Griffioen, Gert Jan Fleuren, Arko Gorter, Ekaterina S Jordanova

**Affiliations:** Department of Pathology, Leiden University Medical Center, Albinusdreef 2, 2333 ZA Leiden, The Netherlands; Department of Medical Statistics and Bioinformatics, Leiden University Medical Center, Leiden, The Netherlands; Angiogenesis Laboratory, Department of Medical Oncology, VU University Medical Center, De Boelelaan 1118, 1081HV Amsterdam, The Netherlands; Department of Gynaecology, Leiden University Medical Center, Leiden, The Netherlands; Center for Gynecological Oncology Amsterdam, VU University Medical Center, Amsterdam, The Netherlands

**Keywords:** Uterine cervical cancer, Tumour microenvironment, Immune response, Angiogenesis, *IL5*, *IL6*, *IL17*, *VEGFA*

## Abstract

**Background:**

The tumour microenvironment comprises a network of immune response and vascularization factors. From this network, we identified immunological and vascularization gene expression clusters and the correlations between the clusters. We subsequently determined which factors were correlated with patient survival in cervical carcinoma.

**Methods:**

The expression of 42 genes was investigated in 52 fresh frozen squamous cervical cancer samples by qRT-PCR. Weighted gene co-expression network analysis and mixed-model analyses were performed to identify gene expression clusters. Correlations and survival analyses were further studied at expression cluster and single gene level.

**Results:**

We identified four immune response clusters: ‘T cells’ (*CD3E*/*CD8A*/*TBX21*/*IFNG*/*FOXP3*/*IDO1*), ‘Macrophages’ (*CD4*/*CD14*/*CD163*), ‘Th2’ (*IL4*/*IL5*/*IL13*/*IL12*) and ‘Inflammation’ (*IL6*/*IL1B*/*IL8*/*IL23*/*IL10*/*ARG1*) and two vascularization clusters: ‘Angiogenesis’ (*VEGFA*/*FLT1*/*ANGPT2*/ *PGF*/*ICAM1*) and ‘Vessel maturation’ (*PECAM1*/*VCAM1*/*ANGPT1*/*SELE*/*KDR*/*LGALS9*). The ‘T cells’ module was correlated with all modules except for ‘Inflammation’, while ‘Inflammation’ was most significantly correlated with ‘Angiogenesis’ (p < 0.001). High expression of the ‘T cells’ cluster was correlated with earlier TNM stage (p = 0.007). High *CD3E* expression was correlated with improved disease-specific survival (p = 0.022), while high *VEGFA* expression was correlated with poor disease-specific survival (p = 0.032). Independent predictors of poor disease-specific survival were *IL6* (hazard ratio = 2.3, p = 0.011) and a high *IL6*/*IL17* ratio combined with low *IL5* expression (hazard ratio = 4.2, p = 0.010).

**Conclusions:**

‘Inflammation’ marker *IL6*, especially in combination with low levels of *IL5* and *IL17*, was correlated with poor survival. This suggests that *IL6* promotes tumour growth, which may be suppressed by a Th17 and Th2 response. Measuring *IL6, IL5* and *IL17* expression may improve the accuracy of predicting prognosis in cervical cancer.

## Background

Cervical cancer is caused by a persistent infection with human papillomavirus (HPV) and represents the second leading cause of cancer-associated deaths worldwide among young women [1]. Infection with HPV initiates an immune response that can generally clear the infection. In some cases the infection can lead to chronic inflammation, which may provide growth signals and support carcinogenesis [2]. Once a tumour has been established, the type of immune response present in the microenvironment is thought to be important for clinical outcome.

Tumour infiltrating T lymphocytes have been shown to be an independent predictor for survival in ovarian and colorectal cancer [3,4]. T lymphocytes can be subdivided in different populations, including cytotoxic CD8^+^ T lymphocytes (CTL) and CD4^+^ T helper 1 (Th1), Th2, Th17 and regulatory T cells (Tregs). CTL and Th1 cells are generally appreciated for their potential to induce or stimulate a specific tumour suppressing immune response. Synthetic long-peptide vaccination in women with HPV16^+^ high-grade vulvar intraepithelial neoplasia has been shown to induce CD4^+^ T helper and CD8^+^ CTL responses, which were correlated with tumour regression [5]. In cervical cancer, we have previously shown that a low number of CTL combined with a high number of Tregs is an independent predictor for poor survival [6]. Since Tregs can control the activity of other T cells, these cells may dampen both a tumour suppressing and a tumour promoting immune response. Indeed, Tregs have been found to be correlated with less invasion in thyroid cancer and improved recurrence-free survival in head and neck cancer [7,8]. A Th2-induced immune response has also been shown to support cervical cancer progression [9]. The role of Th17 cells in cancer is still unclear, as they are capable of inducing both tumour growth and tumour regression [10].

The innate immune system also plays an important role in cervical cancer progression. Our group has shown that mature CD14^+^CD163^−^ M1 type macrophages are an independent predictor for improved survival [11]. CD163^+^ M2 type macrophages have been correlated with poor survival, although the results of different studies are not consistent [12,13]. Tumour associated neutrophils are a heterogeneous cell population associated with poor outcome in different types of cancer [14].

Another important factor for an adequate immune response is the vascular system, which delivers nutrients, but also enables immune cells to enter the tumour site. There appears to be an inverse relationship between new vessel formation (angiogenesis), which supports tumour progression, and vessel adhesiveness (maturation), supporting infiltration of immune cells in the tumour tissue [15]. Angiogenesis is induced by growth factors or cytokines such as vascular endothelial growth factor A (VEGFA), angiopoietin-2 (ANGPT2), fibroblast growth factors (FGFs) and interleukin-8 (IL-8) [15,16]. Angiogenesis, as represented by a high number of blood vessels, has been associated with poor survival in cervical cancer [17]. Vessel maturation, on the other hand, is characterized by signalling and adhesion proteins including vascular cell adhesion molecule-1 (VCAM1), intercellular adhesion molecule-1 (ICAM1), E-selectin (SELE) and ANGPT1 [15,16].

In the present study, we identified combinations of immunological and vascular factors (expression clusters) in cervical carcinoma and determined the correlations between the different clusters, prominent genes and their correlations with clinico-pathological parameters and patient survival. We found clusters characterizing a ‘T cells’, ‘Macrophages’, ‘Th2’, ‘Inflammation’, ‘Angiogenesis’ and ‘Vessel maturation’ pathway. The ‘T cells’ cluster was correlated with all clusters except for ‘Inflammation’, while ‘Inflammation’ was most significantly correlated with ‘Angiogenesis’. T cell infiltration was correlated with improved survival, while ‘Inflammation’ marker *IL6* and ‘Angiogenesis’ marker *VEGFA* were significantly correlated with poor disease specific survival, the former especially when combined with low levels of *IL17* and *IL5*. This suggests that *IL6* promotes tumour growth, which may be suppressed by a Th17 and Th2 response.

## Results

### Gene clustering

**Figure 1:**
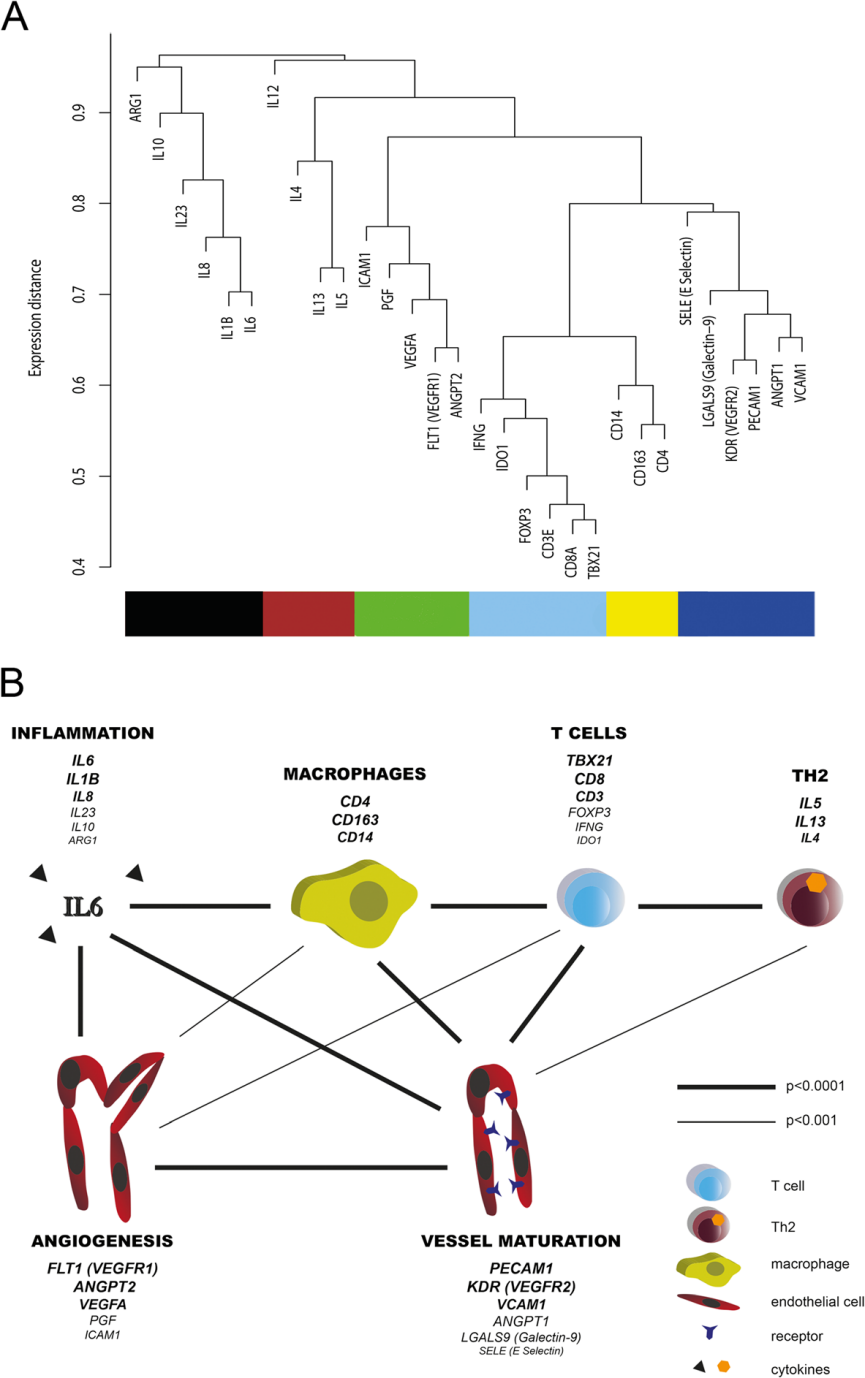
**Gene expression clusters**. WGCNA was performed to detect gene expression clusters, as shown in Figure 1**A**. Expression clusters are indicated by colour bars below the genes that are included in the different clusters. The smallest gene expression distances are present between the genes at the bottom of the figure, representing strong associations. Frequently used gene synonyms are shown between brackets. The correlations between the clusters were tested by mixed model analyses, represented in Figure 1**B**. Only correlations with p < 0.003 are shown, based on the Bonferroni correction for multiple testing.

We investigated the expression of 27 immune response and 15 vascularization marker genes in 52 squamous cervical cancer samples by weighted gene co-expression network analysis (WGCNA), a method developed for network analysis of gene expression data [18]. A gene expression cluster is composed of genes with similar expression patterns. Genes that were not included in a cluster due to lack of correlation with other genes were removed from cluster analysis, including *bFGF*, *FUT9*, *LGALS1*, *LGALS3*, *GATA3*, *IL17A*, *IL17F*, *IL2*, *NE*, *RORC*, *TGFB1* and *TGFB3*, resulting in the dendrogram shown in Figure 1A. Of note, the neutrophil markers *NE* and *FUT9* were expressed at very low levels and were not detected in 26 and 34 out of 52 samples, respectively. *IL17A* was also generally expressed at lower levels than the other genes measured.

Small gene expression distances represent strong correlations. The strongest correlations were found between the T cell markers, for instance between *TBX21* and *CD3E* (r = 0.939, p < 0.0001). Additional clustering was found for the T cell (*CD3E*), CTL (*CD8A*), Th1 (*TBX21*, *IFNG*) and Treg (*FOXP3*, *IDO1*) markers. This gene cluster is referred to as the ‘T cells’ cluster. A ‘Th2’ cluster was represented by the expression of *IL4*, *IL5*, *IL13* (Th2 markers) and *IL12* (Th1 marker). As the expression distance between *IL12* and the other markers was high, *IL12* only weakly correlated with the Th2 markers. The expression of *CD4*, *CD14* and *CD163* represented a ‘Macrophage’ cluster. The last immune response cluster consisted of genes representative of an ‘Inflammation’ response, including *IL1B*, *IL6*, *IL8*, *IL10, IL23* and *ARG1*. In this cluster, *IL10* and *ARG1* were weakly correlated. The ‘Angiogenesis’ cluster included the genes *ANGPT2*, *FLT1* (encoding VEGFR1*)*, *VEGFA*, *PGF* and *ICAM1*, while the ‘Vessel maturation’ cluster included the genes *PECAM1*, *KDR* (encoding VEGFR2*)*, *ANGPT1*, *VCAM1*, *LGALS9* and *SELE* (encoding E-Selectin).

To analyse the correlations between the different gene clusters, we performed mixed model analyses, summarized in Figure 1B. All gene clusters significantly correlated with ‘Vessel maturation’, while the ‘T cells’ cluster significantly correlated with all other clusters except for ‘Inflammation’ (all p < 0.001). The ‘Inflammation’ cluster significantly correlated with the ‘Vessel maturation’, ‘Angiogenesis’ and ‘Macrophages’ clusters (all p < 0.0001), although the individual ‘Inflammation’ genes did not significantly correlate with any of the individual ‘Macrophages’ genes.

In order to study the relationships of more distant genes, we also studied their correlation with separate genes. Extending on the ´Inflammation´ associations, expression of ´Inflammation´ marker *IL6* was inversely correlated with ‘Vessel maturation’ marker *VCAM1* (r = −0.340, p = 0.021). The ‘Inflammation’ and ‘T cells’ clusters were not significantly correlated, but the ‘Inflammation’ marker gene *IL1B* negatively correlated with *CD3E* (r = −0.384, p = 0.009), *CD8* (r = −0.384, p = 0.009), *FOXP3* (r = −0.307, p = 0.031) and *TBX21* expression (r = −0.307, p = 0.032). Expression of the ‘Th2’ cluster gene *IL12* was distant from other ‘Th2’ genes, and only significantly correlated with *IL4* expression (r = 0.506, p = 0.001). Similarly, *ARG1* expression only weakly correlated with *IL10* (r = 0.423, p = 0.004) and *IL23* expression (r = 0.400, p = 0.007), but not with the other genes in the cluster.

### Correlations between gene expression and clinico-pathological parameters

**Figure 2:**
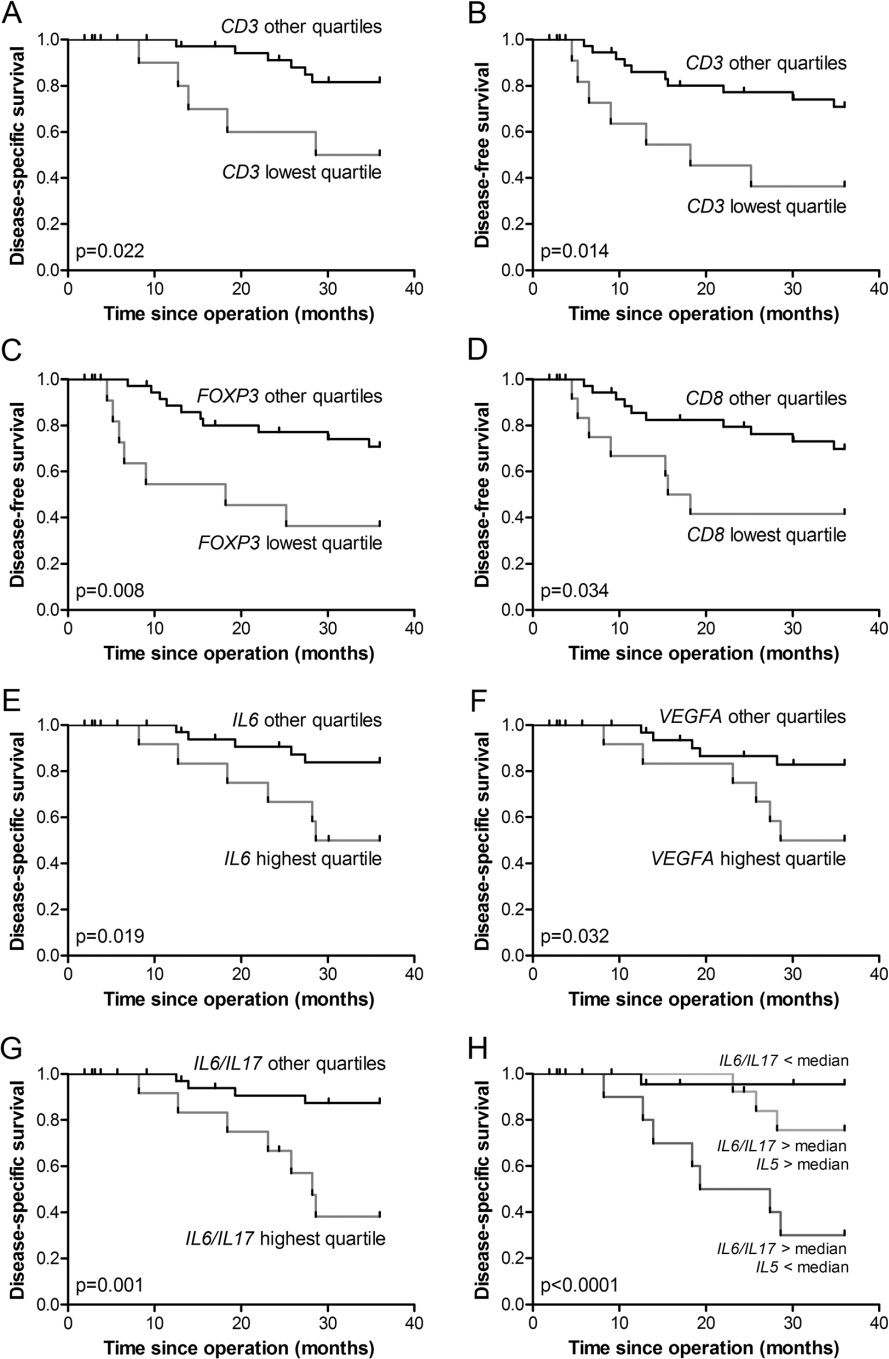
**Survival curves for gene expression.** Kaplan-Meier survival curves for gene expression divided in quartiles. Disease-specific survival for the quartile group with lowest *CD3E* expression compared with the other groups is shown in **A**. Disease-free survival with the same division for *CD3E*, *FOXP3* and *CD8* expression is shown in **B**, **C** and **D**. Disease-specific survival for the quartile groups with highest *IL6* and *VEGFA* expression compared with the rest are shown in **E** and **F**. Disease-specific survival for the quartile group with the highest ratio *IL6*/*IL17* expression compared with the rest is shown in **G**. The combination of an above median *IL6*/*IL17* ratio combined with a below median level of *IL5* compared with the presence of above median expression of *IL5* or relatively low *IL6* expression is shown in **H**.

Correlations between the gene cluster first principal components and the individual genes and clinico-pathological parameters were investigated. Increased expression of the ‘T cells’ cluster significantly was correlated with early tumour node metastasis (TNM) staging (r = 0.37, p = 0.007; see Table 1). This could mainly be attributed to the expression of *FOXP3* (r = 0.39, p = 0.004) and *CD3E* (r = 0.37, p = 0.006). Low expression of *CD3E* was significantly correlated with poor disease-specific survival (p = 0.022; Figure 2A). For disease-free survival, in addition to low *CD3E* expression, low expression of both *FOXP3* and *CD8* were correlated with poor outcome (p = 0.014, p = 0.008 and p = 0.034, respectively; Figures 2B-D). High expression of the ‘Inflammation’ gene cluster was significantly correlated with the absence of vaso-invasion (r = 0.38, p = 0.005), mainly as a result of the contribution of *IL6* (p = 0.002). High expression of *IL6* was significantly correlated with poor disease-specific survival (p = 0.019; Figure 2E).

**Table 1:** **Correlations between clusters and clinico-pathological parameters**

**Clinico-pathological parameter**	**Cluster**	**Correlation (r)**	**p value**	**Gene name**	**Correlation**	**Cq mean (SEM)**	**p value**
TNM	T cells	0.37	0.007	*FOXP3*	0.388		0.004
				*CD3E*	0.374		0.006
Vaso-invasion	Inflammation	0.38	0.005	*IL6*			0.002
					absent	−0.486 (0.193)	
					present	0.348 (0.173)	
Lymph node metastasis				*LGALS9*			0.008
					negative	0.277 (0.166)	
					positive	−0.492 (0.220)	
				*IL2*			0.003
					negative	0.306 (0.176)	
					positive	−0.518 (0.171)	

Correlations between normalized Cq values of cluster first principal components or separate genes and clinico-pathological parameters with p < 0.008 are shown (for the correlation between *LGALS9* and lymph nodes p = 0.0077). The correlations between single gene expression and TNM stage were tested by the Pearson correlation test and since increased Cq values represent decreased expression, an inverse correlation was found. The correlations for separate genes and vaso-invasion or lymph nodes were tested using independent samples t-tests. Mean normalized Cq values of the gene expression within a category are given to indicate the direction of correlation (inverse for vaso-invasion).

Expression of the ‘Vessel maturation’ marker *LGALS9* was correlated with lymph node metastasis (p = 0.008). This association was also observed for the expression of *IL2* (p = 0.003).

Within the ‘Angiogenesis’ cluster, high expression of *VEGFA* (highest quartile) was significantly correlated with poor disease-specific survival (p = 0.032; Figure 2F).

### Hazard ratios for independent prognostic factors

We investigated which genes were independent prognostic factors for survival using a multivariate analysis with one representative gene per cluster (Table 2). High *IL6* expression was the best predictor of poor disease-specific survival with a hazard ratio of 2.2 (p = 0.002). After correction for clinico-pathological parameters, the hazard ratio of *IL6* expression was 2.3 (p = 0.011). IL-6 was a signature cytokine of the ‘Inflammation’ gene cluster, which included cytokines that are often associated with the induction of an IL-17 response: IL-1β, IL-6, IL-8 and IL-23 [19]. Studying the correlation between IL-17 and survival, we found that high expression of *IL17* showed a trend toward an association with improved survival (p = 0.087). Since *IL6* and *IL17* expression were not correlated in our study, we studied whether the ratio of *IL6* and *IL17* has an association with survival. Indeed, a high ratio of *IL6* over *IL17* expression was significantly correlated with poor survival (p = 0.001; Figure 2G). Since the intratumoural immune response in cervical cancer is dominated by Th1, Th2, Th17 and Treg cells, we further analysed the contribution of the Th1 marker *TBX21* and Th2 marker *IL5*. In order to maintain sufficient group sizes for this analysis, groups were divided based on the median expression level. An above median *IL6* over *IL17* ratio remained significant (p = 0.004). We did not observe a significant effect for the addition of *TBX21*, which was significantly correlated with Th1, Treg and CTL markers. In contrast, the combination of above median *IL6* relative to *IL17* expression combined with a below median level of *IL5* was significantly correlated with a worse prognosis (p < 0.0001; Figure 2H). This combination was shown to be an independent predictor for poor prognosis corrected for lymph node status, tumour size and infiltration depth with a hazard ratio of 4.2 (p = 0.010; Table 2). For both multivariate Cox regression analyses, the results remained similar and significant upon adding postoperative therapy as a parameter.

**Table 2:** **Cox regression analyses**

	**Univariate Cox regression**		**Multivariate Cox regression**			
**Variable**	**Hazard ratio (95% CI)**	**p value**	**Hazard ratio (95% CI)**	**p value**	**Hazard ratio (95% CI)**	**p value**
Lymph node status	1.20 (0.35-4.11)	0.769	1.56 (0.30-8.07)	0.599	1.35 (0.27-6.77)	0.718
Tumour size	1.05 (1.02-1.09)	0.006	1.05 (0.99-1.11)	0.121	1.06 1.00-1.12)	0.073
Vaso-invasion	1.22 (0.36-4.15)	0.756				
Infiltration depth	1.06 (0.99-1.13)	0.096	1.01 (0.92-1.11)	0.785	0.97 (0.89-1.06)	0.556
*IL6*	2.21 (1.34-3.66)	0.002	2.29 (1.21-4.34)	0.011		
*IL5*	0.96 (0.61-1.51)	0.853				
*ANGPT2*	1.12 (0.56-2.24)	0.746				
*TBX21*	0.997 (0.53-1.87)	0.993				
*CD14*	0.45 (0.17-1.18)	0.104				
*PECAM1*	0.90 (0.46-1.76)	0.754				
*IL6/IL17 + IL5*	4.66 (1.90-11.41)	0.001			4.17 (1.41-12.40)	0.010

Univariate Cox regression hazard ratios are shown for the critical prognostic categorical clinico-pathological parameter lymph node tumour positivity and the continuous variables tumour size (per mm) and infiltration depth (per mm), as well as for the expression of genes representative for the different clusters. Normalized Cq values were converted to expression values to obtain hazard ratio’s corresponding with increased presence of the gene product. Multivariate Cox regression analyses are shown for the genes significant in the univariate analysis combined with the most critical clinico-pathological parameters (restricted by the number of patients). The combination of the *IL6*/*IL17* ratio and *IL5* is divided in three categories: a low ratio, a high ratio and high *IL5* levels and a high ratio combined with low *IL5* levels.

### Correlation between RNA and protein expression

**Figure 3:**
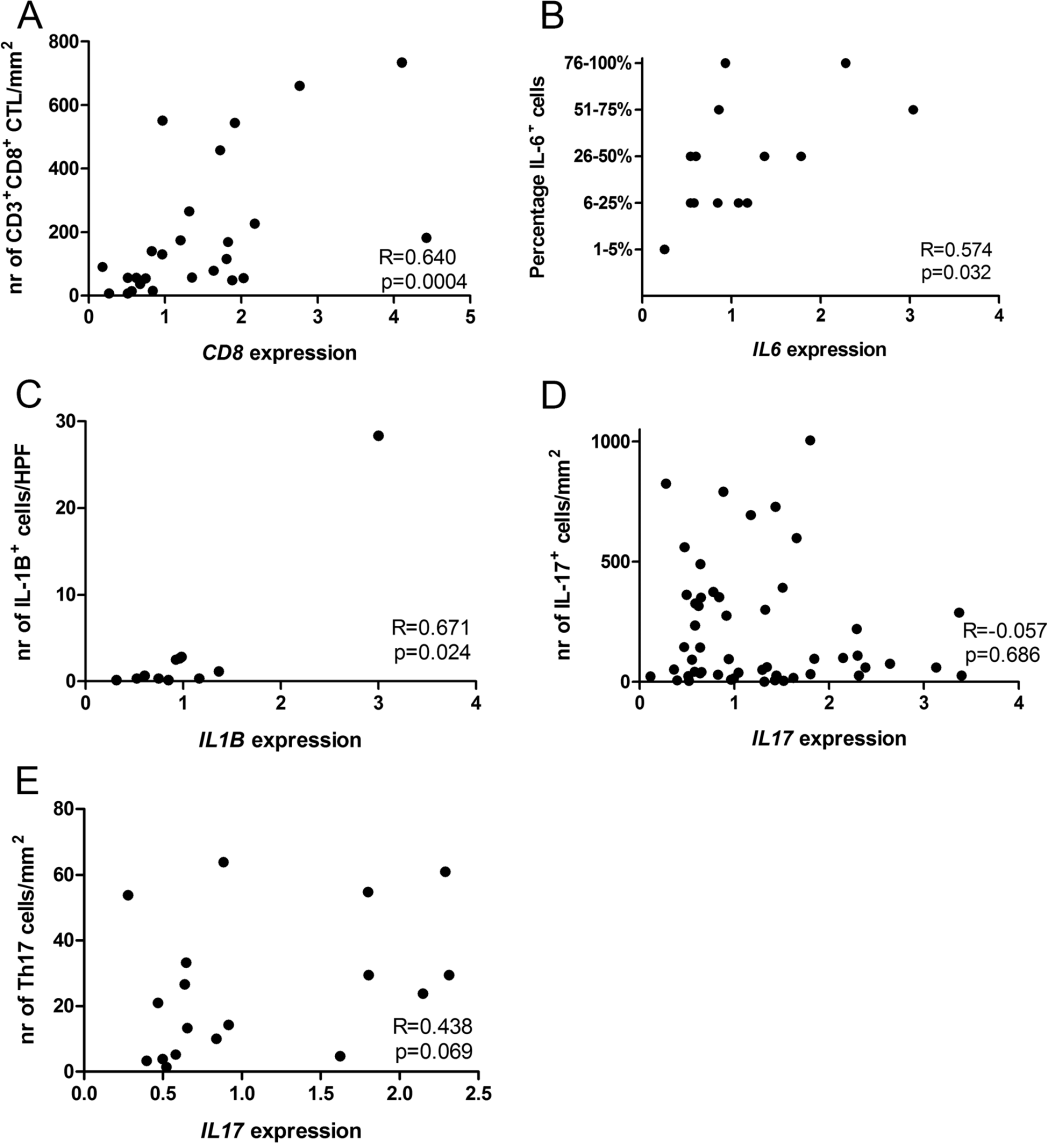
**Correlation between RNA and protein expression.** The RNA expression levels determined by qRT-PCR in fresh frozen tissue were compared with the number of cells expressing the corresponding protein as determined by IHC. Correlations were determined for *CD8* expression and the number of CD3^+^CD8^+^ cells **(A)**, *IL6* and the percentage of IL-6^+^ cells **(B)**, *IL1B* and the number of IL-1β expressing cells **(C)**, *IL17* and the total number of IL-17^+^ cells **(D)** and Th17 cells **(E)**.

We studied whether the expression levels of some of the most relevant genes determined by qRT-PCR on fresh frozen tissue correlated with the number of cells expressing the corresponding proteins determined by IHC in FFPE tissue. The RNA expression level of CD8 was significantly correlated with the number of CD3^+^CD8^+^ cells (r = −0.640, p = 0.0004; Figure 3A). The expression of *IL6* and the percentage of IL-6^+^ cells were also significantly correlated (r = 0.574; p = 0.032; Figure 3B). The number of cells expressing IL-1β was significantly correlated with *IL1B* RNA expression (r = 0.628; p = 0.029; Figure 3C).

We further studied whether *IL17* RNA expression was correlated with the number of IL-17^+^ cells, mainly comprising neutrophils, or specifically with Th17 cells. There was no significant correlation between *IL17A* RNA expression and the total number of IL-17^+^ cells (r = −0.048, p = 0.737; Figure 3D), but there was a trend toward a positive correlation with the number of IL-17^+^CD3^+^ Th17 cells (r = −0.438, p = 0.069; Figure 3E).

## Discussion

In the present study, we used WGCNA to identify gene expression clusters associated with immune response and vessel formation processes in cervical carcinoma. Six gene expression clusters were identified: ‘T cells’, ‘Macrophages’, ‘Th2’, ‘Inflammation’, ‘Vessel maturation’ and ‘Angiogenesis’. The clusters were named according to the pathways the genes are involved in, but may be expressed and induced by tumour epithelial or infiltrating immune cells, or both. High expression of the ‘T cells’ cluster was associated with early TNM staging, and low expression of *CD3E*, *CD8* and *FOXP3* were correlated with poor disease-specific and disease-free survival. This supports earlier observations where the absence of a lymphocytic infiltrate was shown to be a predictor of poor survival [20]. The ‘T cells’ cluster also showed strong correlations between the expression of CTL, Th1 and Treg marker genes. In agreement with these observations, we and others have previously shown that the number of FoxP3^+^ Tregs strongly correlates with the number of tumour infiltrating T cells [6,8]. In contrast, we have shown before that a high number of FoxP3^+^ Tregs scored specifically within the tumour epithelium, especially relative to the number of CD8^+^ CTL, was correlated with poor survival, an observation also made in other tumour types [20-22]. Collectively, these data indicate that T cell infiltration is correlated with improved survival, whereas a relatively high number of Tregs, specifically within the tumour epithelium, counteracts the tumour suppressing immune response.

The ‘Th2’ gene expression cluster was characterized by *IL4*, *IL5*, *IL13* and *IL12* expression. Surprisingly, expression of transcription factor *GATA3* was not correlated with the expression of any of these genes, suggesting that *GATA3* RNA expression may not be suitable marker for the Th2 response. Although a Th2 response is regarded as immunosuppressive in cervical cancer [9], HPV-specific T cells associated with regression of high-grade VIN lesions, have been shown to produce high levels of both IFNy and IL-5 [5]. In agreement with the latter observation, low *IL5* levels were an indicator of poor prognosis in combination with high *IL6* relative to *IL17* levels.

The ‘Inflammation’ gene expression cluster was not significantly correlated with the ‘Th2’ and ‘T cells’ clusters, indicating that these clusters represent distinct pathways. High expression of *IL6* represented a dominant ‘Inflammation’ response and was significantly correlated with poor disease-specific survival. A high number of stromal IL-6^+^ cells was previously shown to be correlated with poor disease-specific survival in an overlapping patient cohort [23] and other types of cancer [24]. IL-6 might drive STAT3 expression in tumour cells [25], induce angiogenesis and epithelial-mesenchymal transition [26,27] and induce differentiation of dendritic cells and macrophages toward tumour promoting cells [28,29]. Additionally, *IL6* was correlated with the absence of vaso-invasion. We have observed a significant correlation between IL-17 and IL-1β and the absence of vaso-invasion by IHC as well ([30] and unpublished data). *IL1β*, also a member of the ‘Inflammation’ gene cluster, showed a trend toward a correlation with the absence of vaso-invasion. These results suggest that this type of inflammatory response may prevent metastatic spread of the tumour cells via the blood or lymphatic vasculature.

The ‘Inflammation’ gene cluster was characterized by cytokines that are often associated with the induction of an IL-17 response: IL-1β, IL-6, IL-8 and IL-23 [19]. However, *IL17* expression was not significantly correlated with the ‘Inflammation’ cluster, T-cell or neutrophil related genes. Mature neutrophils have been shown to express no or very low mRNA levels for granule proteins [31]. In our study, both *IL17* and neutrophil markers *NE* and *FUT9* were expressed at very low levels. Since we have shown by IHC that IL-17 is mainly expressed by neutrophils in cervical cancer [30], this suggests that *IL17A* RNA expression is primarily derived from Th17 cells. The absence of a correlation with T cell markers and the ‘Inflammation’ gene cluster is likely due to the small size of the Th17 population.

While a qRT-PCR analysis by Tosolini et al. showed that a Th1 cluster and high *FOXP3* expression were correlated with improved disease-free survival in colon cancer, corresponding with our results, this group also found a correlation between a high Th1/Th17 gene cluster ratio and improved disease-free survival [32]. In the current work, the ‘Inflammation’ gene cluster was more important for patient survival. To study whether *IL17* might have an opposite effect on survival compared to *IL6*, the ratio of *IL6* and *IL17* was analysed. Indeed, this ratio was significantly correlated with poor survival, corresponding with previous observations that a high number of Th17 cells is correlated with improved disease-specific survival in cervical carcinoma [30]. Since the immune response in cervical cancer is predominantly characterized by Th1, Th2, Th17 and Treg cells, the contributions of the ‘T cells’ signature marker *TBX21* and the ‘Th2’ signature marker *IL5* were studied. We did not observe a significant association with the Th1 marker *TBX21*, which is supposed to be critical for an tumour suppressing immune response [5]. Strikingly, the most prognostic independent risk factor was a high *IL6* over *IL17* ratio combined with a low expression level of Th2 marker *IL5*, with a hazard ratio of 4.2 (p = 0.010). Since we did not find a correlation between *IL5* and *IL6* expression in cervical cancer, this suggests that the effect of a high amount of *IL6* is dampened by a Th2 response.

‘Vessel maturation’ adhesion markers were correlated with *KDR* encoded VEGFR2 expression, which has been described before [33] and suggests that VEGFR2 might primarily be involved in vessel maturation in cervical cancer. Expression of the ‘Vessel maturation’ marker *LGALS9* was correlated with tumour positive lymph nodes, suggesting it might play a role in metastasis to the lymph nodes.

The ‘Inflammation’ cluster was most significantly correlated with the ‘Angiogenesis’ cluster. Although the ‘Angiogenesis’ cluster marker ICAM1 is an adhesion protein, VEGFA has been described to first induce ICAM1 expression to prepare endothelial cells for migration, after which ICAM1 is downregulated [34]. The ‘Angiogenesis’ marker *VEGFA* was significantly correlated with poor disease-specific survival (p = 0.032), which is in agreement with our previous study where we showed that *VEGFA* expression correlates with the number of blood vessels in cervical cancer, in its turn correlated with poor disease-free survival [35]. Correspondingly, Yuan et al. showed that both RNA and protein expression of *VEGFA* were correlated with poor survival in non-small-cell lung cancer [36].

Although IL-17 has been reported to induce vascularization via VEGF-dependent and -independent mechanisms in cancer [37,38], we did not find a significant association between *IL17* and angiogenesis or vessel maturation. Correspondingly, we did not find a correlation between the number of IL-17^+^ cells and the number of CD105^+^ vessels in a series of 151 squamous cervical carcinoma samples (data not shown).

## Conclusions

**Figure 4:**
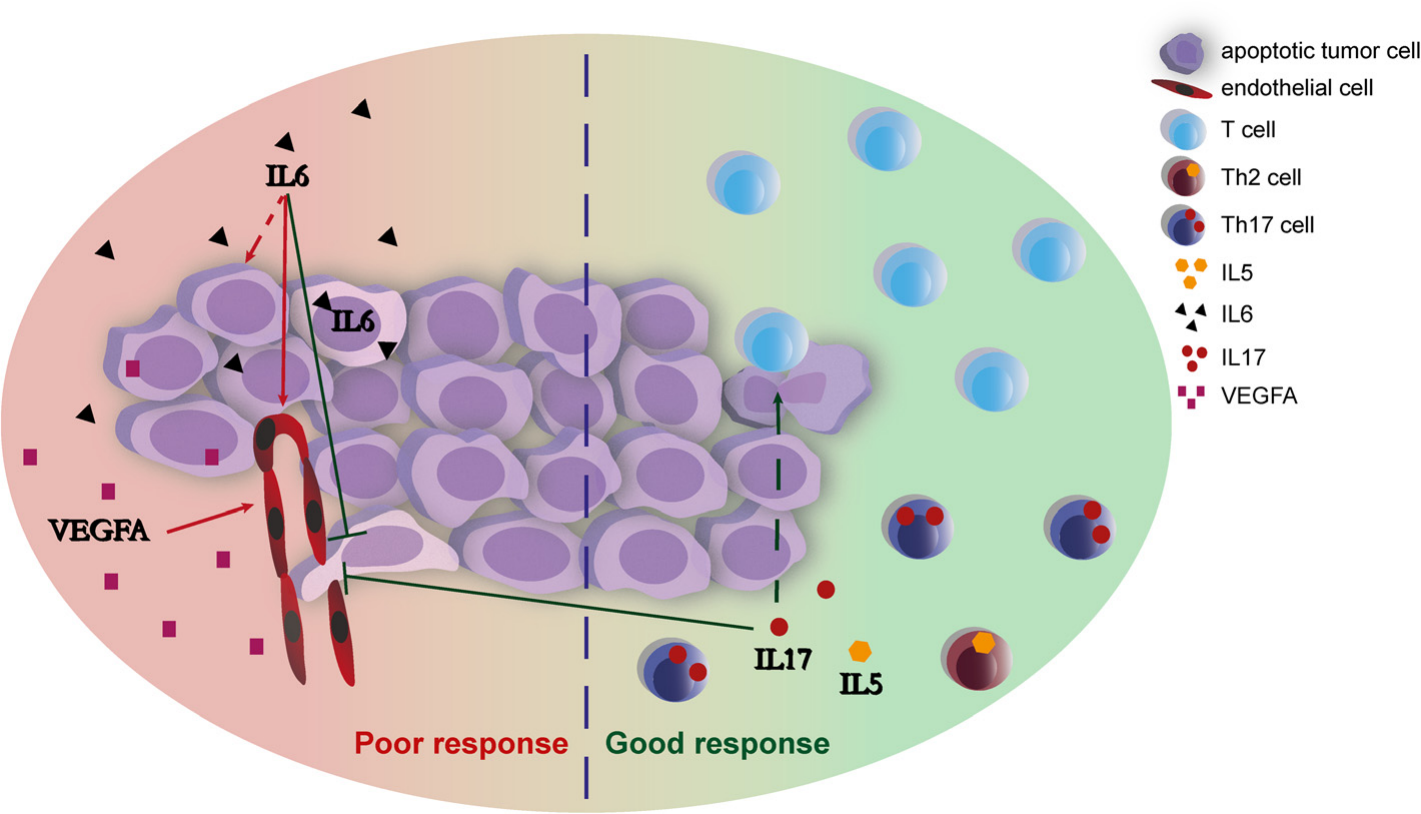
**Th17 counteracts the effect of IL6.** Of the 27 immunological and 15 vascularization markers analysed, *CD3*, *IL5*, *IL6*, *IL17A* and *VEGFA* were most significantly correlated with the clinical outcome of cervical cancer patients. A ‘T cells’ response, indicating the presence of Th1, CTL and Treg cells and represented by blue cells, was correlated with improved prognosis. *IL6* and *VEGFA*, the latter indicative of angiogenesis, were both correlated with poor prognosis. *IL6*, expressed by both tumour cells and infiltrating immune cells, may directly promote tumour growth, indicated by the interrupted arrow. High *IL17* (Th17) and *IL5* (Th2) expression were correlated with improved prognosis, specifically relative to *IL6* expression. *IL6* expression was also correlated with the absence of vaso-invasion, indicated by the blunted arrow on the tumour cell entering the vessel. We have also shown this correlation before for IL-17 protein expression [30], suggesting that the IL-6/IL-17 pathway may prevent tumour spread via the blood or lymphatic vasculature.

By using a qRT-PCR array, we identified *CD3E*, *IL6*, *VEGFA* and a high *IL6*/*IL17* ratio combined with low *IL5* expression as the most prognostic factors in squamous cervical cancer. While high expression of T cell markers was correlated with improved prognosis, and high expression of angiogenesis marker *VEGFA* was correlated with poor prognosis, *IL17* expressed by Th17 cells could counteract the tumour promoting effects of *IL6*, even more so combined with a Th2 response characterized by *IL5*. A proposed model of the factors most relevant for disease outcome is shown in Figure 4. Measuring *IL6,* especially in combination with *IL5* and *IL17* expression may improve the accuracy of predicting prognosis. Moreover, it supports the development of combined anti-IL-6 and anti-VEGF therapies. Since we have found correlations between ‘Inflammation’ markers and the absence of vaso-invasion, blocking IL-6 might increase the risk of tumour cell invasion. Since VEGFA expression has been correlated with tumour invasiveness [39,40], and the presence of vaso-invasion negatively affects clinical outcome, blocking both IL-6 and VEGFA has the potential to counteract both tumour growth and invasion.

## Materials and methods

### Patient material

Fresh frozen squamous cervical cancer specimens from patients who underwent primary surgical treatment for cervical cancer between 1989 and 2005 with sufficient material available for analysis were obtained from the biobank of the departments of Pathology and Gynecology from the Leiden University Medical Center (the Netherlands). None of the patients had received preoperative therapy and follow-up data were obtained from patient medical records. Samples containing 50-90% tumour epithelial cells and no uninvolved normal tissue were selected by staining a 4 μm section with haematoxylin and eosin (n = 56). Median follow-up time was 6.2 years. Patient and tumour characteristics are listed in Table 3. Samples were handled according to the medical ethical guidelines described in the Code of Conduct for Proper Secondary Use of Human Tissue of the Dutch Federation of Biomedical Scientific Societies. Patients receive information on the secondary use of the tissue sampled for diagnostic use and can actively object to secondary use. According to the guidelines, all human material used in this study has been anonymised. Because of the anonymisation, retrospective research does not require ethical approval from the Institutional Review Board and individual consent is not required.

**Table 3:** **Patient characteristics**

**Clinico-pathological parameter**	**Category**	**N = 52 (%)**
**Age**	Median	43
	Range	24-77
**FIGO stage** ^**1,2**^	IB	41 (79)
	IIA	10 (19)
**TNM stage**	IB1	16 (31)
	IB	2 (4)
	IB2	19 (37)
	IIA	8 (15)
	IIB	5 (10)
	IIIA	0 (0)
	IIIB	1 (2)
	IV	1 (2)
**Lymph nodes**	Negative	33 (63)
	Positive	19 (37)
**Tumour size (mm)** ^**2**^	<40	20 (38)
	≥40	29 (56)
**Vaso-invasion** ^**2**^	Absent	22 (42)
	Present	29 (56)
**Infiltration depth (mm)** ^**2**^	<15	26 (50)
	≥15	22 (42)
**HPV type**	16	31 (60)
	18	11 (21)
	Other	10 (19)

^1^FIGO, International Federation of Gynecologists and Obstetricians.

^2^Data were not available for all patients.

### RNA isolation and quality control

RNA was isolated from four 20 μm slides using Trizol (Life Technologies, Carlsbad, USA) and DNase treated and purified using RNeasy Mini columns (Qiagen, Hilden, Germany). The RNA integrity and quantity were analysed using RNA 6000 Nano chips in a 2100 Bioanalyzer (Agilent Technologies, Santa Clara, USA). Samples with RIN < 5 were excluded from further analysis (n = 4), because the Cq values of the reference genes were disproportionally low. The median RIN value was 7.9.

### qRT-PCR array immune response markers

Genomic DNA removal, cDNA synthesis, pre-amplification and qRT-PCR were performed using RT^2^ Profiler PCR Arrays (Qiagen) according to the manufacturers’ instructions. In brief, 300 ng RNA was treated with DNA elimination mix and cDNA was subsequently synthesized. The intended PCR products were preamplified, followed by Side Reaction Reducer and heat inactivation. A Sybr Green-based qRT-PCR reaction was performed in duplicate on a CFX384 system (Bio-Rad, Hercules, USA). A custom combination of primer sets was used to analyse different T cell and macrophage markers: *ARG1*, *CD14*, *CD163*, *CD3E*, *CD4*, *CD8A*, *FOXP3*, *GATA3*, *IDO1*, *IFNG*, *IL1B*, *IL10*, *IL12A*, *IL13*, *IL17A*, *IL17F*, *IL2*, *IL23A*, *IL4*, *IL5*, *IL6*, *RORC*, T-bet encoding *TBX21*, *TGFB1* and *TGFB3*. From the four reference genes included based on reported stability in cervical cancer tissue, the most stably expressed genes *EEF1A1* and *RPLP0* were used for normalization. Averaged duplicate measurements were scaled by standard deviation. Negative genomic DNA contamination and positive reverse transcriptase and PCR controls were included for each sample.

### cDNA synthesis and qRT-PCR IL-17A and neutrophil markers

Since *IL17A* expression measured by the RT^2^ Profiler PCR Array was low for all samples and not detected in eight samples, an additional qRT-PCR with Primer-BLAST [41] designed primers for *IL17A* was performed on all samples. To further complement the assay, qRT-PCRs were performed for the neutrophil markers fucosyltransferase 9 (*FUT9*) and neutrophil elastase (*NE*). cDNA was synthesized as described previously [42]. Sybr Green-based qRT-PCR was performed in duplicate using 1:125 diluted cDNA and 3 pmol primers. Primers and annealing temperatures used were for *IL17A*: forward 5′-CCCCCGGACTGTGATGGTCAAC-3′ and reverse GCGGCACTTTGCCTCCCAGAT at 56.7°C, *FUT9*: forward 5′- AGGCCACCCTTCCAGAAATG-3′ and reverse 5′- TGCTTGGCACTTCAAACACG-3′ at 64.5°C and *NE*: forward 5′- ATTCTCCAGCTCAACGGGTC-3′ and reverse 5′- GATTAGCCCGTTGCAGACCA-3′ at 63.8°C. The primer products were validated by sequencing. Reference genes *EEF1A1* and *RPLP0* were quantified using 4 pmol RT^2^ qPCR primer assays (Qiagen) at an annealing temperature of 60°C as per the manufacturers’ instructions. Replacing the cDNA template by milliQ was used as negative control. The qRT-PCR, normalization and scaling were performed as described for the RT^2^ Profiler PCR array. Since RNA expression was detected in all samples by the Primer-BLAST primers, all *IL17A* analyses were performed with the *IL17A* expression measured by the Primer-BLAST primers.

### cDNA synthesis and qRT-PCR vascularization markers

Vascularization markers *ANGPT1*, *ANGPT2*, basic fibroblast growth factor (*bFGF*), platelet endothelial cell adhesion molecule (*PECAM1*), placental growth factor (*PGF*), *IL8*, Galectin-1 (*LGALS1*), Galectin-3 (*LGALS3*), Galectin-9 (*LGALS9*), *VEGFA*, VEGFR1 encoding fms-related tyrosine kinase 1 (*FLT1*), VEGFR2 encoding kinase insert domain receptor (*KDR*), *ICAM1*, *VCAM1* and *SELE* were measured on all samples, except for samples containing less than 100 ng RNA per μl (n = 2). cDNA synthesis, primers and qPCR conditions were as described before [43,44]. Reference genes, normalization and scaling were performed as described for the immune response markers.

### Immunohistochemistry

In previous studies, immunohistochemistry (IHC) has been performed on at least ten formalin-fixed, paraffin-embedded (FFPE) samples corresponding to the fresh frozen samples used in this study. Briefly, CD3^+^CD8^+^ CTL were stained by a mixture of rabbit anti-CD3 (Abcam, Cambridge, UK), mouse IgG2b anti-CD8 (Novocastra, Newcastle, UK) and mouse IgM anti-CD57 (developed in-house), followed by goat anti-rabbit IgG-A546, goat anti-mouse IgG2b-A647 and goat anti-mouse IgM-A488 (Invitrogen, Life Technologies, Carlsbad, USA) [6]. Fifteen images per slide were obtained in 26 samples using an LSM510 confocal laser scanning microscope equipped with a PH2 Plan-NEOFluar 25x/0.80 oil objective (Zeiss, Göttingen, Germany) in a multitrack setting. The number of IL-6^+^ cells was scored in 14 samples stained with rabbit anti-IL-6 (Abcam) followed by biotinylated swine anti-rabbit (Dako, Glostrup, Denmark) and biotinylated HRP-streptavidin (Dako) [23]. The percentage of positive cells was counted in six random high-power fields. The number of IL-1β^+^ cells was scored in 12 overlapping samples stained with goat anti-IL-1β (R&D Systems) and the goat HRP-polymer kit [23]. Positive cells were counted in the tumour stroma of six random high-power fields. IL-17 was stained on all 52 samples by goat anti-IL-17 (R&D Systems, Abingdon, UK) followed by goat HRP-polymer (Biocare Medical, Concord, USA) [30]. Cells were digitally scored in 4–6 random images at a 200x magnification. CD3^+^IL-17^+^ cells were stained by a mixture of mouse IgG1 anti-CD3 (Dako) and goat anti-IL-17 followed by rat anti-mouse IgG1-AP (Southern Biotech, Birmingham, USA) and donkey anti-goat-HRP (Abcam). Six random images were taken in 18 samples using a DM4000B spectral microscope equipped with a HC Plan APO 20x objective (Leica Microscopy CMS GmbH, Wetzlar, Germany).

### Statistical analysis

WGCNA was performed using R version 3.0.2 [45]. Weights were computed using a power of 3 to obtain the best combination of power, scale-free topology and connectivity values. Average linkage hierarchical clustering was performed. The distance between subclusters was determined by using the average distances between all potential gene pairs. Small clusters were only assigned to clusters belonging to the same branch. Gene expression clusters were summarized using the first principal components.

To test for correlations between gene expression clusters and genes, mixed model analyses were performed using SPSS version 20.0 (IBM Corp., Armonk, USA). All combinations between the six expression clusters were tested for correlations with a Bonferroni corrected significance level of p < 0.003. A cutoff for single gene correlations (r) of 0.3 and significance level of p < 0.05 were used. Correlations between RNA expression and IHC data was tested using the Spearman’s rank correlation rho test. A p value < 0.05 was considered statistically significant.

Correlations between gene expression clusters or separate genes and clinico-pathological variables were tested using the independent samples t-test and Wilcoxon Mann–Whitney U test for categorical variables and the Pearson and Spearman’s rank correlation rho for continuous variables. Six gene expression clusters were tested for each parameter with a Bonferroni corrected significance level of p < 0.008.

Normalized Cq values were converted to expression values to obtain correlations corresponding with increased presence of the gene product for Kaplan-Meier and Cox proportional hazards survival models. For Kaplan-Meier curve generation and log rank analyses, gene expression levels were divided in four equal quartiles and the lowest quartile (low expression) or highest quartile (high expression) was compared with the other quartiles.

## Abbreviations

ANGPT: Angiopoietin
CTL: Cytotoxic T lymphocytes
FGF: Fibroblast growth factor
FIGO: International Federation of Gynecologists and Obstetricians
FUT: Fucosyltransferase
HPV: Human papillomavirus
ICAM: Intercellular adhesion molecule
IHC: Immunohistochemistry
IL: Interleukin
NE: Neutrophil elastase
Th1: T helper 1
TNM: Tumour node metastasis
Tregs: Regulatory T cells
VCAM: Vascular cell adhesion molecule
VEGF: Vascular endothelial growth factor
WGCNA: Weighted gene co-expression network analysis
